# Supplementary material for: The interactome of intact mitochondria by cross-linking mass spectrometry provides evidence for coexisting respiratory supercomplexes
Source: Mol Cell Proteomics. 2017 Dec 8;17(2):216–32. doi: 10.1074/mcp.RA117.000470 (PMC5795388; doi:10.1074/mcp.RA117.000470)
Supplement: Supplemental Data [file supp_RA117.000470_133922_0_supp_23978_fzffwf.zip › spectra_annotation/mito_DR_spectra_annotation/420-1-6-1-11-1.pdf]

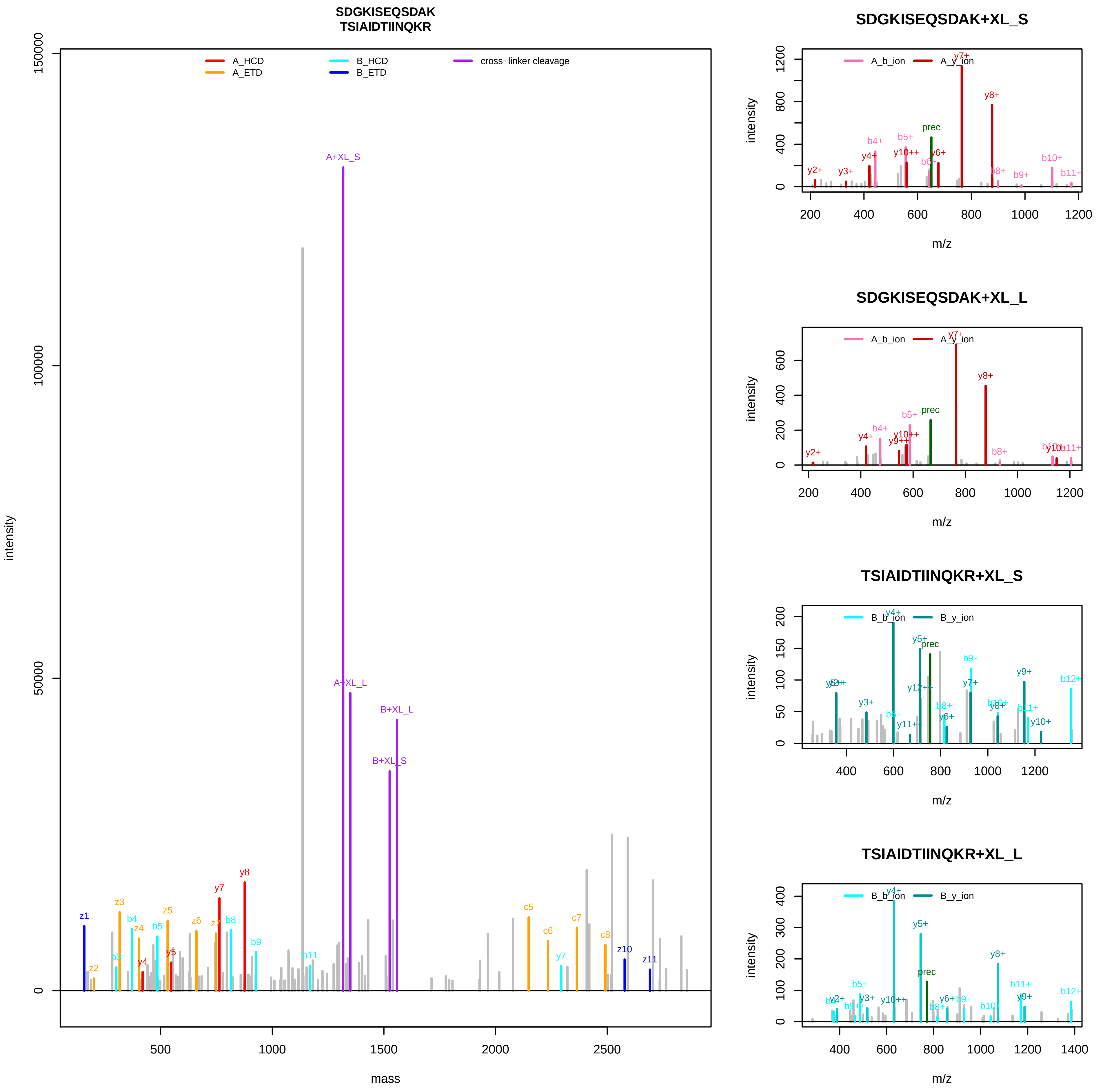
SDGKISEQSDAK
TSIAIDTIINQKR
A_HCD
A_ETD
B_HCD
B_ETD
cross−linker cleavage
A+XL_S
A+XL_L
B+XL_L
B+XL_S
z1
z2
z3
b3
b4
z4
y4
b5
z5
y5
z6
z7
y7
b8
y8
b9
b11
c5
c6
y7
c7
c8
z10
z11
intensity
mass
0
50000
100000
150000
500
1000
1500
2000
2500
SDGKISEQSDAK+XL_S
A_b_ion
A_y_ion
prec
m/z
SDGKISEQSDAK+XL_L
TSIAIDTIINQKR+XL_S
B_b_ion
B_y_ion
TSIAIDTIINQKR+XL_L
